# White-Light-Emitting Supramolecular Polymer Gel Based on *β*-CD and NDI Host-Guest Inclusion Complex

**DOI:** 10.3390/polym13162762

**Published:** 2021-08-17

**Authors:** Srayoshi Roy Chowdhury, Sujay Kumar Nandi, Sahabaj Mondal, Santosh Kumar, Debasish Haldar

**Affiliations:** Centre for Advance Functional Materials, Department of Chemical Sciences, Indian Institute of Science Education and Research Kolkata, Mohanpur 741246, India; srayoshi05@gmail.com (S.R.C.); nandisujay457@gmail.com (S.K.N.); mondalsahabaj7@gmail.com (S.M.); sk13ms033@gmail.com (S.K.)

**Keywords:** supramolecular polymer, gel, *β*-CD, NDI, host-guest complex

## Abstract

Supramolecular polymer formed by non-covalent interactions between complementary building blocks entraps solvents and develops supramolecular polymer gel. A supramolecular polymer gel was prepared by the heating-cooling cycle of *β*-cyclodextrin (*β*-CD) and naphthalenedimide (NDI) solution in N,N-dimethylformamide (DMF). The host-guest inclusion complex of *β*-CD and NDI **1** containing dodecyl amine forms the supramolecular polymer and gel in DMF. However, *β*-CD and NDI **2,** having glutamic acid, fail to form the supramolecular polymer and gel under the same condition. X-ray crystallography shows that the alkyl chains of NDI **1** are complementary to the hydrophobic cavity of the two *β*-CD units. From rheology, the storage modulus was approximately 1.5 orders of magnitude larger than the loss modulus, which indicates the physical crosslink and elastic nature of the thermo-responsive gel. FE-SEM images of the supramolecular polymer gel exhibit flake-like morphology and a dense flake network. The flakes developed from the assembly of smaller rods. Photophysical studies show that the host-guest complex formation and gelation have significantly enhanced emission intensity with a new hump at 550 nm. Upon excitation by a 366 nm UV-light, NDI **1** and *β*-CD gel in DMF shows white light emission. The gel has the potential for the fabrication of organic electronic devices.

## 1. Introduction

Recognition, combination, and repeated arrays of complementary monomeric building blocks connected by highly directional and reversible non-covalent interactions yield supramolecular polymers [1,2,3]. Supramolecular polymers and gels have potential due to their wide applications in various fields such as optoelectronics [4,5], delivery vehicles [6,7], tissue repairing and engineering [8,9,10,11], and stimuli-responsive materials [12,13,14]. Supramolecular polymers are formed by highly directional and reversible non-covalent interactions such as hydrogen bonding, π-π stacking, Van der Waals interaction and hydrophobic interactions [15,16,17,18]. Though there are many reports on supramolecular polymer gel [19], the fabrication of a supramolecular polymer and gel from π-conjugated semiconductor systems is still challenging due to their projected applications in photovoltaics, electronic device, organic light-emitting diodes and field-effect transistors.

Due to their potential application in organic electronic devices, the developments of white-light-emitting organic materials are highly important [20,21,22,23]. There are a large number of examples of white light-emitting materials from supramolecular polymers [24,25], organic-inorganic hybrid materials [26,27,28], π-conjugated systems [29] and single molecules [30,31,32]. Huang and co-workers prepared white-light-emitting fluorescent polymeric materials resulting from the aggregation of a single fluorescent chromophore through intermolecular quadruple hydrogen bonding [33]. Banerjee and co-workers developed peptide-based white light-emitting systems containing two components [34]. We are looking for supramolecular polymers by reversible non-covalent interactions of chromophores and study the effect of the host-guest complex formation on optical properties. Previously, advances in the knowledge of white light-emitting systems were made [35,36]; herein we developed a supramolecular polymer gel by a heating-cooling cycle of *β*-cyclodextrin (*β*-CD) and chromophore naphthalenedimide (NDI) solution in N,N-dimethylformamide (DMF). The host-guest inclusion complex between *β*-CD and NDI **1** containing dodecyl amine results in the supramolecular polymer and gel in DMF. However, *β*-CD and NDI **2,** having glutamic acid, fail to form the supramolecular polymer and gel under the same condition. X-ray crystallography shows that the alkyl chains of NDI **1** are complementary to the hydrophobic cavity of the two *β*-CD units. From rheology, the storage modulus is approximately 1.5 orders of magnitude larger than the loss modulus, which indicates the physical crosslink and elastic nature of the thermo-responsive gel. From FE-SEM studies, the supramolecular polymer gel exhibits flake-like morphology and a dense flake network. The flakes have developed from the assembly of smaller rods. Photophysical studies depict that the host-guest complex formation and gelation have significantly enhanced emission intensity with a new hump at 550 nm. Upon excitation by a 366 nm UV-light, NDI **1** and *β*-CD gel in DMF shows white-light emission.

## 2. Materials and Methods

### 2.1. Synthesis of NDI ***1*** and NDI ***2***

The NDI **1** was synthesized by dissolving naphthalenetetracarboxylic dianhydride and dodecylamine in dry DMF; it was then refluxed for 6 h with around 50% yield [37]. The NDI **2** was synthesized by dissolving naphthalenetetracarboxylic dianhydride (5 mmol) and glutamic acid in DMF and reflux for 24 h in ca. 30% yields. The mostly transparent solution was concentrated under reduced pressure and 50 mL of DCM was added. For every case, the organic layer was washed with 2 M HCl (3 × 50 mL), brine (2 × 50 mL) and dried over anhydrous Na_2_SO_4_, and evaporated under vacuum to obtain the corresponding NDI derivatives. The crude product was purified by silica gel using hexane: ethyl acetate (1:2) as an eluent.

### 2.2. NMR Experiments

All NMR experiments were performed on a 400 MHz spectrometer (Jeol, Tohoku, Japan) and 500 MHz spectrometer (Bruker, Zurich, Switzerland) at 298 K. Compound concentrations were in the range 1–10 mM in CDCl_3_ and DMSO-*d*_6._

### 2.3. FT-IR Spectroscopy

Solid-state FT-IR spectra following the KBr disk technique were measured in a Spectrum RX1 spectrophotometer (Perkin Elmer, Waltham, MA, USA).

### 2.4. Mass Spectrometry

Mass spectra of the compounds were recorded on a Q-Tof Micro YA263 high-resolution mass spectrometer (Waters Corporation, Milford, MA, USA) by positive-mode electrospray ionization.

### 2.5. UV/Vis Spectroscopy

The absorption spectra of the samples were recorded on a Perkin Elmer UV-Vis spectrophotometer (Perkin Elmer, Waltham, MA, USA).

### 2.6. Fluorescence Spectroscopy

All fluorescence spectra were recorded on a Fluoromax 3 spectrometer (HORIBA, Kyoto, Japan) using a 1 cm path length quartz cell. Slit widths 5/5 were used.

### 2.7. Polarised Optical Microscope

A small amount of gel/solution of the compound was placed on a clean glass cover slip, then dried by slow evaporation, and then visualized at 40×magnification optical microscope equipped with polarizer and CCD camera (Olympus, Shinjuku City, Tokyo, Japan).

### 2.8. Field Emission Scanning Electron Microscopy

Field emission-scanning electron microscopy (FE-SEM) was used to know the morphologies of the reported compounds. For supramolecular gel, a thin gel slice was placed on a clean microscopic glass slide and dried by slow evaporation. For NDI solutions, a drop of sample solution was placed on a clean microscopic glass slide and dried by slow evaporation. The materials were gold-coated, and the micrographs were taken in an FE-SEM apparatus (JEOL Scanning Microscope-JSM-6700F, (Jeol, Tohoku, Japan).

### 2.9. Rheology Experiments

To understand the mechanical strengths, we performed rheological measurements on a MCR 102 rheometer (Anton Paar, Garz, Austria) using a steel parallel plate geometry with an 8 mm diameter at 25 °C. To control the temperature accurately during the experiment, the rheometer wasattached to a Peltier circulator thermo cube (Anton Paar, Garz, Austria). The storage modulus (G′) and loss modulus (G″) of the hydrogels were recorded using that experimental setup.

### 2.10. Single Crystal X-ray Diffraction Study

Single-crystal X-ray analysis of NDI **2** was recorded on a Bruker high-resolution X-ray diffractometer instrument (Bruker, Zurich, Switzerland) with MoKα radiation. Data were processed using the Bruker SAINT package (Bruker, Zurich, Switzerland) and the structure solution and refinement procedures were performed using SHELX97 (Bruker, Zurich, Switzerland). CCDC 2093019 contains the crystallographic data for NDI **2**.

Crystallographic data of NDI **2**: space group P 21/c, a = 9.0701(2), b =14.3646(3), c = 14.4885(4) Å, α = 90°, β = 89.932(2)°, γ = 90°,V = 1934.59(8) Å^3^, Z = 2, dx =1.440 gcm^−3^, T = 107 K, R1 0.0434 and wR2 0.1124 for 3370 data with I > 2σ(I).

### 2.11. Fluorescence Lifetime Imaging Microscopy (FLIM)

The FLIM images were obtained by a confocal laser scanning microscope (Axio Observer A1, (Becker & Hickl Berlin, Germany) from ZEISS coupled with a DCS-120 system from Becker & Hickl (Becker & Hickl Berlin, Germany) GmbH for fluorescence lifetime imaging and a picosecond diode laser (BDL-488-SMC, (Becker & Hickl Berlin, Germany) with λ_ex_ = 488 nm. A BH GVD-120 scan controller controlled the scanning. The BH HPM-100-40 hybrid detector module in the DCS-120 system was controlled by DCC-100 software (Becker & Hickl Berlin, Germany). A long-pass filter (HQ495LP) was placed to block the excitation light, and a narrow band-pass filter of 525–550 nm (HQ525/50) was used to monitor the emission. The TCSPC FLIM system is controlled by the “SPCM” TCSPC operating software (Becker & Hickl Berlin, Germany) and the DCC-100 detector controller software. The emitted light from a selected point of the sample was collected by the microscope lens, scanned by the galvanometer mirrors, separated from the excitation beam, split into two channels of different wavelengths, and focused into pinholes in a plane conjugate with the focal plane in the sample. Out-of-focus light was thus suppressed. The FLIM images were analyzed in SPC-Image software (Becker & Hickl Berlin, Germany) for decay measurement at a particular point of the sample.

## 3. Results and Discussions

### 3.1. Design and Synthesis

**Figure 1:**
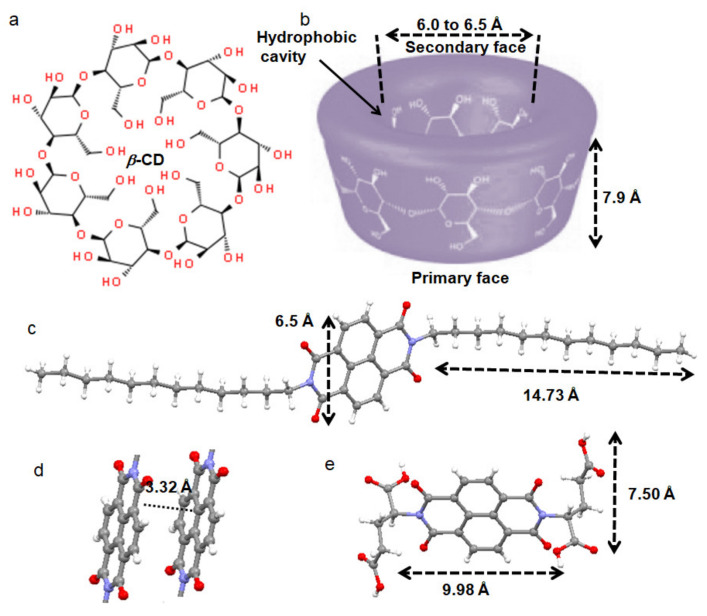
(**a**)The chemical structure of *β*-CD; (**b**) The graphic showing the 3D shape and cavity of *β*-CD; (**c**) The solid-state structure of NDI **1**; (**d**) The face to face π-π stacking interaction between the naphthalenediimide central core of NDI **1**; (**e**) The solid-state structure of NDI **2**.

We are looking for supramolecular polymers by molecular recognition, combination, and repeated arrays of complementary monomeric building blocks connected by reversible non-covalent interactions; we then study the optical properties. For that purpose, the chromophores naphthalenedimides (NDI) **1** and **2** (Figure 1) were synthesized by solution reaction, purified, and characterized by ^1^H NMR, ^13^C NMR, FT-IR, and mass spectrometry analysis (Supporting Information Figures S1–S15 and Scheme S1). The design principle behind this was to develop the supramolecular polymer by the host-guest inclusion complex of *β*-CD and chromophore NDI. *β*-CD is a macrocyclic oligosaccharide of seven glucose subunits connected by α-1,4 glycosidic bonds (Figure 1a). It has a toroidal shape with a primary face (smaller opening) and a secondary face (larger opening). The toroid is 7.9 Å thick and has a cavity of diameter ca. 6.0–6.5 Å (Figure 1b). The *β*-CD can host hydrophobic molecules inside the cavity. The exterior of the toroid is hydrophilic which makes *β*-CD highly soluble in water but not soluble in organic solvents. NDI **1** contains two hydrophobic dodecyl amine groups (Figure 1c). The alkyl chains of dodecyl amines (lengths 14.73 Å) can accommodate inside *β*-CD cavities and form a host-guest inclusion complex. We have also designed and synthesized NDI **2** with two glutamic acids (Figure 1e), assuming that due to four hydrophilic acid functional groups at both terminal positions, it will not form a host-guest complex and supramolecular polymer with *β*-CD.

### 3.2. X-ray Structures of the Building Blocks

The NDI **1** and **2** were further characterized by X-ray crystallography. Crystals suitable for X-ray crystallography were obtained from methanol (NDI **1**) and DMSO (NDI **2**) solution by slow evaporation. The X-ray analyses reveal that the NDI **1** adopts an extended structure with a completely planar naphthalenediimide core (Figure 1c) [37]. Methylene groups of side chains are in anti-conformations (Figure 1c). The length of the dodecyl alkyl chain is 14.73 Å which is about 2 times that of *β*-CD thickness. The length of the NDI core is 9.98 Å and 6.5 Å wide; that means that the NDI core cannot be accommodated inside the *β*-CD cavity. At higher order, molecules of NDI **1** self-assemble by face-to-face π–π interactions between the central naphthalenediimide moiety (inter planar C–C distance is 3.38 Å) (ESI, Figure S1). The shortest distance between two NDI **1** cores is 3.32 Å (Figure 1d). From X-ray crystallography, the glutamic acid side chains are in the trans-position of the naphthalenediimide core (Figure 1e). The length of the glutamic acid side chain is 7.50 Å, which is perpendicular to the NDI core (9.98 Å long, 6.5 Å wide). Hence, due to the large size and hydrophilic acid groups, the NDI **2** cannot form a host-guest complex with the *β*-CD. The π-π stacking interaction between adjacent NDI **2** molecules is also absent there. The shortest distance between two NDI **2** cores is 8.24 Å (ESI, Figure S2). In crystal packing, the NDI **2** molecules are stabilized by multiple hydrogen bonding, C–H…π interactions, and oxygen-π interactions (ESI, Figure S3).

### 3.3. Gelation Study

**Figure 2:**
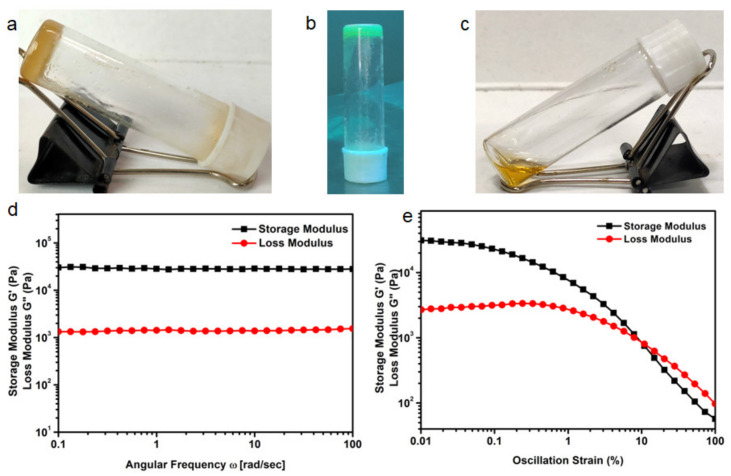
(**a**) The image showing NDI **1** and *β*-CD gel in DMF; (**b**) The NDI **1** and *β*-CD gel in DMF under 254 nm light showing blue-green emission; (**c**) The image showing NDI **2** and *β*-CD failed to form a gel in DMF; (**d**) Effect of angular frequency on NDI **1** and *β*-CD gel in DMF at 25 °C; (**e**) Effect of strain on NDI **1** and *β*-CD gel in DMF at 25 °C.

As the NDI **1** contains hydrophobic chains, we thought that the host-guest interactions with the *β*-CD can significantly modulate the self-assembly behavior of the compounds and help in supramolecular polymer formation and gelation. *β*-CD is soluble in limited solvents like water, DMF, and DMSO. First, we studied the gelation in water. Though NDI **1** is insoluble in water, in the presence of *β*-CD it forms a white suspension in water. Even after several heating-cooling cycles and sonication, it fails to form a gel in water. An amount of 6.0 mg of NDI **1** and 11.0 mg of *β*-CD (1:1 ratio) were placed in DMF (0.5 mL) in a small vial, the vial closed, heated, and shaken for 30 seconds to solubilize the NDI **1** and *β*-CD. After standing for 5 minutes, a yellow opaque gel was formed. The gel is thermo-responsive (ESI, Figure S4) and the assembly of the building blocks can change reversibly in response to temperature. The gel transition temperature (T_g_) was determined at this concentration by gradually heating the vial containing the gel in an oil bath. At 66°C the gel turned into a solution. This thermal sol-gel-sol cycle was performed a second time, and a gel was obtained again. Gel formation was confirmed by the inverted vial test (Figure 2a). The DMF gel shows a greenish colour under 254 nm light (Figure 2b), however, the NDI **2** with *β*-CD fails to form a gel under the same condition (Figure 2c). We also tested that individually NDI **1** and *β*-CD do not form a gel in DMF even at high concentrations. This indicates that the gel formed due to host-guest complex formation and development of supramolecular polymer matrix. The gel is very stable under the laboratory environment for a couple of months.

### 3.4. Rheology Study

Rheology studies were carried out to examine the mechanical strength of the gel in DMF. Rheology data was taken as a function of angular frequency as well as oscillatory strain. A rheometer (Anton Paar, Modular Compact Rheometer) having a steel parallel plate geometry with an 8 mm diameter with a 0.7 mm gap was used for rheology experiments at 25 °C. In rheology, there are two main parameters, the elastic response, which is measured by storage modulus G′, and the viscous response, which is measured by loss modulus G″, dissipated as heat. In the frequency sweep experiment, the storage modulus G′ is 1.5 orders of magnitude higher than the loss modulus G″ over the entire angular frequency range, which confirms the formation of the gel (Figure 2d). In the amplitude sweep experiment, the loss modulus G″ becomes greater than the storage modulus G′ at 9.6% oscillation strain, which indicates that the gel breaks above this strain (Figure 2e). We conclude that the gel is formed by physical crosslink and is elastic in nature.

### 3.5. Morphology

**Figure 3:**
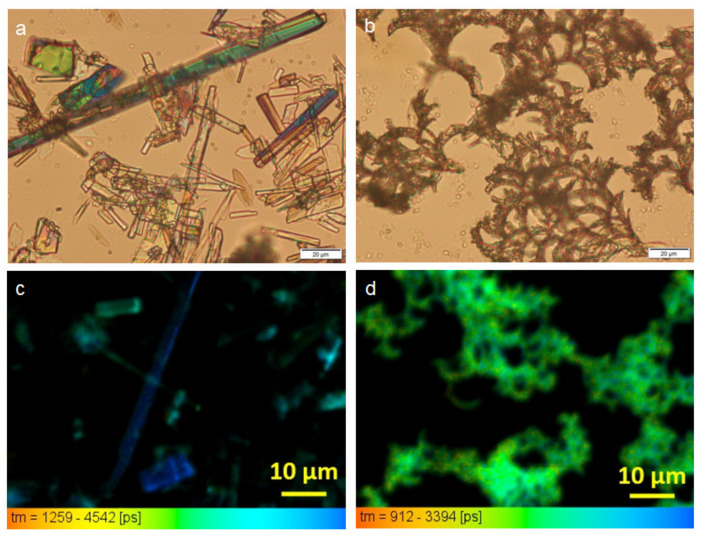
(**a**)The POM image of only NDI **1** in DMF showing blue-green birefringence; (**b**) The POM image of NDI **1** and *β*-CD gel in DMF; (**c**) The FLIM image of only NDI **1** in DMF showing blue fluorescence; (**d**) The FILM image showing NDI **1** and *β*-CD gel in DMF showing greenish-blue emission.

The morphology of the gel was studied by polarized optical microscopy (POM). The sample prepared from only NDI **1** solution in DMF drop casted on a glass slide followed by drying at 30 °C for 48 h shows discrete crystals (Figure 3a) under POM. The crystal shows blue-green birefringence under polarized light (Figure 3a). However, the sample prepared from only *β*-CD solution in DMF and drop casted on a glass slide followed by drying at 30 °C for 48 h shows monodisperse fiber-like morphology (ESI, Figure S5) under POM. The NDI **1** and *β*-CD gel in DMF has significant self-healing nature (ESI, Figure S6). A thin gel slice was observed under POM; after cutting damage spontaneous self-healing occurred, although the damaged area was still slightly visible. A slice of the NDI **1** and *β*-CD gel in DMF was placed on a microscopic glass slide and it was allowed to dry under reduced pressure at room temperature for two days. The POM image of that xerogel depicts entangled fiber morphology (Figure 3b).

Furthermore, we studied the NDI **1** and *β*-CD gel in DMF by the fluorescence lifetime imaging (FLIM) technique. Simply, fluorescence lifetime is the average time a molecule remains in an excited state before going to the ground state by emitting a photon. Hence, FLIM produces an image based on the differences in the excited state decay rate from a fluorescent sample and does not depend on excitation intensity, absorption by the sample, photo-bleaching, sample thickness, and concentration. Figure 3c shows the FLIM image of NDI **1** in DMF having blue fluorescence. However, on supramolecular polymer formation by NDI **1** and *β*-CD gel in DMF the fluorescence colour changes to greenish-blue (Figure 3d), as depicted by the FLIM technique.

**Figure 4:**
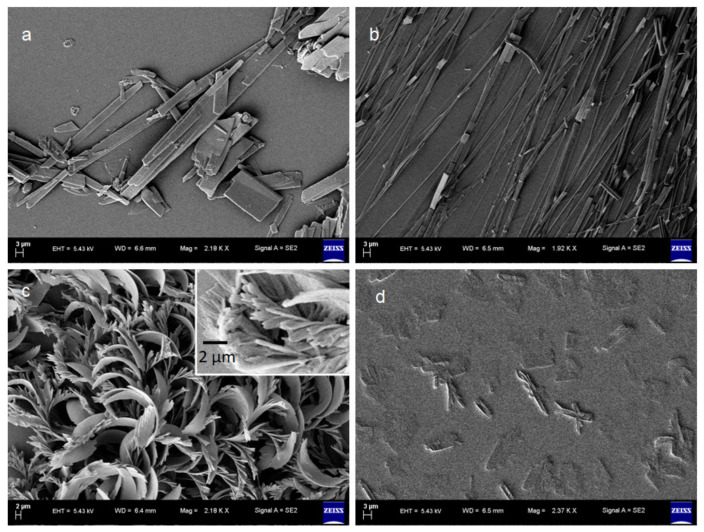
(**a**) The FE-SEM image of only NDI **1** in DMF showing discrete crystals; (**b**) The FE-SEM image of only *β*-CD in DMF showing several micrometers long rod-like structure; (**c**) The FE-SEM image of NDI **1** and *β*-CD xerogel showing flake like entangled morphology. Inset: showing the flakes have developed from the assembly of smaller rods; (**d**) The FE-SEM image of NDI **2** and *β*-CD in DMF solution.

Further, we studied the morphology of the gel by field emission scanning electron microscopy (FE-SEM). From FE-SEM, images of the sample prepared by drop cast of only NDI **1** in DMF solution show the polydisperse discrete crystal morphology (Figure 4a). The sample obtained by drop-casting of only *β*-CD in DMF solution exhibits monodisperse discrete fibers morphology in FE-SEM (Figure 4b). From FE-SEM, xerogels of NDI **1** and *β*-CD obtained from DMF show the entangled flake-like structure of length up to a few micrometers (Figure 4c). The flakes are formed by the assembly of smaller rods (Figure 4c inset). From FE-SEM, images of the sample prepared by drop cast of NDI **2** and *β*-CD in the DMF solution show the existence of different entities (Figure 4d).

### 3.6. Optical Properties

**Figure 5:**
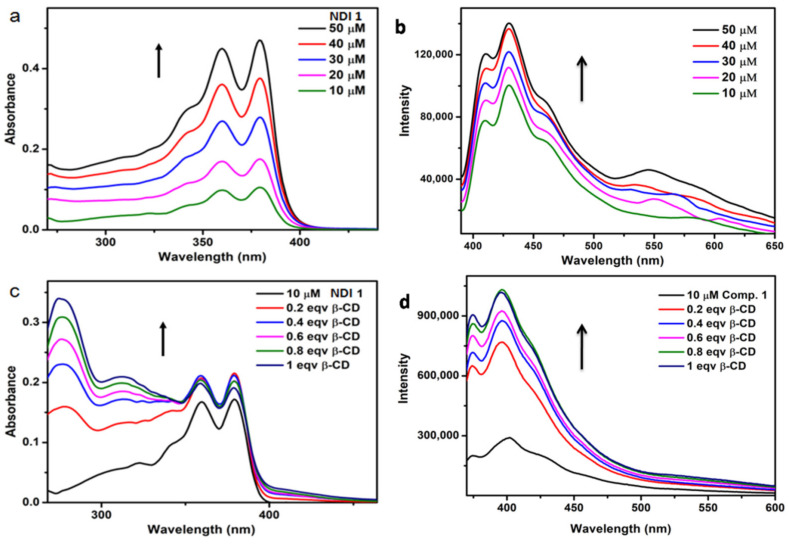
(**a**)Absorption spectra of NDI **1** in DMF with increasing concentration; (**b**) The emission spectra of NDI **1** in DMF with increasing concentration, excitation at 380 nm;(**c**) Absorption spectra of NDI **1** in DMF with the gradual addition of *β*-CD; (**d**) The emission spectra of NDI **1** in DMF with the gradual addition of *β*-CD, excitation at 360 nm.

The aggregation property of NDI **1** was studied by UV-Vis spectroscopy. The typical UV-Vis spectra of NDI **1** show absorption bands at 360 and 380 nm (Figure 5a). With increasing concentration of NDI **1** from 10 to 50 μM, the absorption band intensity also increases, however, there is no change in band position.The typical emission spectra (excitation at 380 nm) show bands at 410 and 430 nm (Figure 5b). With increasing concentration of NDI **1** from 10 to 50 μM, the emission band intensity at 410 and 430 nm also increases without any shifting. However, a rather broad emission band appeared at longer wavelengths (550 nm) because of the formation of excimer-type species at high concentration (Figure 5b) [38]. The host-guest complex formation of NDI **1** and *β*-CD was studied by UV-Vis spectroscopy (Figure 5c). With the addition of 0.2 equivalent of *β*-CD to 10 μM NDI **1** in DMF, the intensities of those bands increased and a new band appeared at 275 nm (Figure 5c). With the gradual addition of *β*-CD, the intensities of the bands increased gradually (Figure 5c). Further, we studied the host-guest complex formation of NDI **1** and *β*-CD in DMF by emission spectroscopy. The typical emission spectra of NDI **1** showed bands at 402 nm on excitation at 360 nm (Figure 5d). With the addition of 0.2 equivalent of *β*-CD the band shifted to 396 nm (Figure 5d) and a new hump appeared at 550 nm, indicating the formation of excimer-type species at this low concentration. With the gradual addition of *β*-CD, the emission intensities of the bands increased gradually (Figure 5d).

**Figure 6:**
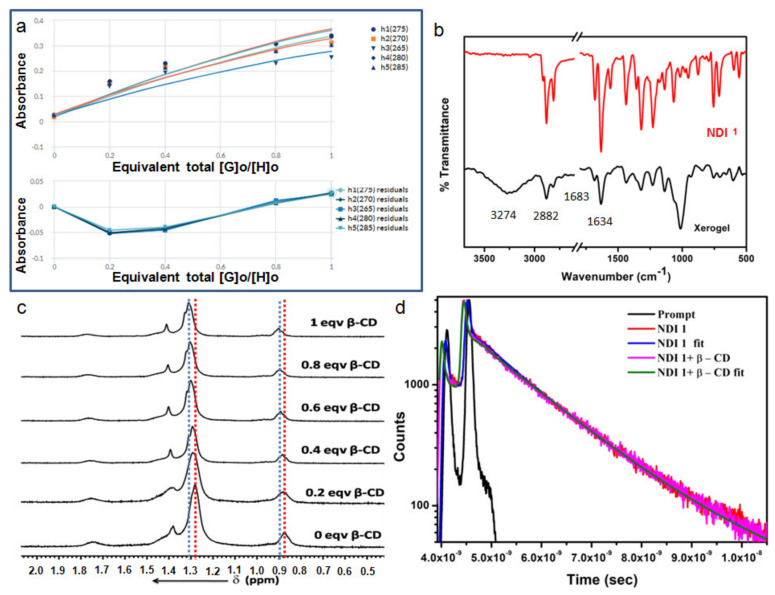
(**a**) The binding stoichiometry of NDI **1** and *β*-CD in DMF by fitting emission data at 275 nm using the bindfit methods, http://supramolecular.org (accessed on 24 July 2021). (**b**) The FT-IR spectra of only NDI **1** (red) and *β*-CD- NDI **1** xerogel from DMF; (**c**) Part of ^1^H NMR spectra of NDI **1** with the gradual addition of *β*-CD in DMF-*d*_7_; (**d**) Time-resolved fluorescence decay traces of only NDI **1** and NDI **1**-*β*-CD complex. Excitation wavelength is 375 nm and the emission wavelength is 428 nm.

Binding stoichiometry is very important in the host-guest complex formation. To determine the binding stoichiometry, we used the bindfit methods [39,40] from http://supramolecular.org/software (accessed on 24 July 2021). The emission data at 275 nm shows the best fit with 1:1 stoichiometry (Figure 6a). We also calculated the binding constant between NDI **1** and *β*-CD, and it was found to be 2.76 × 10^5^ M^−1^ (±27%).

FT-IR spectroscopy following the KBr disk technique was measured to examine the host-guest complex and supramolecular polymer formation by the NDI **1** and *β*-CD in DMF xerogel. The FT-IR spectra of xerogel (dried gel) of NDI **1** and *β*-CD depict a broad signal with a peak at around 3274 cm^−1^, assigned as hydrogen-bonded OH (Figure 6b). The C-H of the dodecyl group appears at 2882 cm^−1^. The carbonyl peak appears at 1634 cm^−1^ (Figure 6b). The broad peak at 1060 cm^−1^ is responsible for secondary cyclic alcohols (Figure 6b). Hence, the NDI **1** and *β*-CD form a hydrogen-bonded structure in xerogel.

Moreover, the formation of supramolecular polymers was further confirmed by ^1^H NMR titration experiments. The ^1^H NMR study of NDI **1** in DMF-*d*_7_ with the gradual addition of *β*-CD shows little downfield shift of the NDI **1** alkyl CH_2_ protons (1.28 to 1.31, Δδ 0.03) and CH_3_ protons (0.88 to 0.90, Δδ 0.02) with growing *β*-CD concentration (Figure 6c), which suggests the interaction between the NDI **1** alkyl chain and *β*-CD. However, the aromatic protons show no shift, indicating no interaction between the NDI **1** aromatic rings and *β*-CD.

Time-resolved fluorescence decay traces show that the average lifetime of NDI **1** is 1.16 ns (Figure 6d). Excitation wavelength is 375 nm and the emission wavelength is 428 nm. From the Figure 6d (lifetime data), the excited-state lifetime changes by the incremental addition of *β*-CD (lifetime is 1.37 ns). This confirms the host-guest complex formation between NDI **1** and *β*-CD. From the above experimental results, we propose that the two dodecyl alkyl chains from two different NDI **1** molecules inserted and collapse inside the cavity of a single *β*-CD. The repeated arrays of these host-guest complexes form a supramolecular polymer that entangled to form the matrix that encapsulated DMF to form a gel (Figure S7) [41].

**Figure 7:**
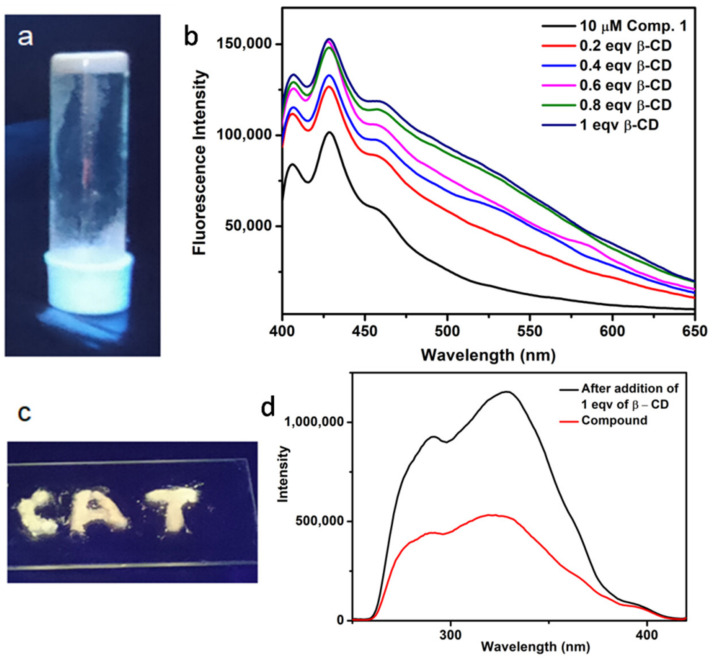
(**a**) The NDI **1** and *β*-CD gel in DMF emits white-light upon excitation by 366 nm UV-light; (**b**) The emission spectra of NDI **1** in DMF with the gradual addition of *β*-CD, excitation at 380 nm; (**c**) The design made by NDI **1** and *β*-CD gel in DMF on glass surface showing white-light emission upon excitation by 366 nm UV-lamp. (**d**) The excitation spectra at this emission.

From the previous report, the supramolecular gels can be used as a light-emitting material [42]. Here, upon excitation at 366 nm, emission of white-light from the NDI **1** and *β*-CD gel in DMF was observed (Figure 7a). Further, we studied this phenomenon by UV-Vis and fluorescence spectroscopy. The emission spectra of NDI **1** show bands at 428 nm on excitation at 380 nm (Figure 7b). With the addition of *β*-CD the emission intensity not only increases 1.5-fold (Figure 7b) but also causes a broad hump to appear at 550 nm. The broad emission band appeared at longer wavelengths due to the formation of excimer-type species [38]. Because of such a heterogeneous nature, the emission spectrum spans almost the entire red–green–blue region and the system emits white-light. The white light-emitting self-healing (ESI, Figure S6) NDI **1** and *β*-CD gel in DMF can be applied on any surface. For this purpose, the glass slide was made by injecting a thin layer of the gel. The resulting layer emits a white-light on irradiation by a 366 nm UV-light (Figure 7c). Figure 7d shows the excitation spectra at this emission.

## 4. Conclusions

In conclusion, a supramolecular polymer gel that emits white-light was developed. The host-guest inclusion complex between *β*-CD and NDI **1** containing dodecyl amine forms the supramolecular polymer as well as a gel in DMF. However, *β*-CD and NDI **2**, having glutamic acid, fails to form the supramolecular polymer and gel under the same condition. From X-ray crystallography, the alkyl chains of NDI **1** are complementary to the hydrophobic cavity of the *β*-CD and the NDI aromatic core is bigger than the *β*-CD cavity. In the case of frequency sweep rheology experiments, the storage modulus (G′) is 1.5 orders greater than the loss modulus (G″) over the entire range of frequency, which indicates the physical crosslink and elastic nature of the thermo-responsive gel. Microscopic images of the supramolecular polymer gel exhibit a dense flake network morphology and the flakes developed from the assembly of smaller rods. Photophysical experiments depict that the host-guest complex formation and gelation have significantly enhanced emission intensity and a new hump appears at 550 nm, because of the formation of excimer-type species. Due to such a heterogeneous nature, the emission spectrum spans almost the entire red–green–blue region. Moreover, the NDI **1** and *β*-CD gel in DMF shows white-light emission upon excitation by a 366 nm UV-lamp. These results will be helpful to tailor organic electronic devices.

## Data Availability

Crystallographic data CCDC 2093019 for NDI **2**.

## Supplementary Materials

The following are available online at https://www.mdpi.com/article/10.3390/polym13162762/s1, Scheme S1: Schematic presentation of NDI **1** and **2**, Figure S1: Higher order crystal structure of NDI **1** showing face-to-face π–π interactions between the central naphthalene diimide moieties, Figure S2: Crystal structure of NDI **2** showing absence of π-π stacking interaction, Figure S3: Packing diagram of NDI **2** along crystallographic b and c direction, Figure S4: The supramolecular gel in DMF exhibit reversible sol to gel transitions induced by temperature at 66 °C, Figure S5: POM image of *β*-CD in DMF, Figure S6: Self healing property of NDI **1** and *β*-CD in DMF gel by POM images, Figure S7: The schematic representation of the supramolecular polymer and gel formation, Figure S8: ^1^H NMR (400 MHz, CDCl3, δ in ppm, 298 K) spectrum of NDI **1**, Figure S9: ^13^C NMR (100 MHz, CDCl3, δ in ppm, 298 K) spectrum of NDI **1**, Figure S10: Mass spectrum of NDI **1**, Figure S11: FT-IR spectrum of NDI **1**, Figure S12: ^1^H NMR (500 MHz, DMSO-d6, δ in ppm, 298 K) spectrum of NDI **2**, Figure S13: ^13^C NMR (125 MHz, DMSO-d6, δ in ppm, 298 K) spectrum of NDI **2**, Figure S14: Mass spectrum of NDI **2**, Figure S15: FT-IR spectrum of NDI **2**.
