# Supplementary material for: White-Light-Emitting Supramolecular Polymer Gel Based on β-CD and NDI Host-Guest Inclusion Complex
Source: Polymers (Basel). 2021 Aug 17;13(16):2762. doi: 10.3390/polym13162762 (PMC8400613; doi:10.3390/polym13162762)
Supplement: Supplementary file 1 [file polymers-13-02762-s001.zip › polymers-1301756-supplementary.pdf]

Supporting Information

White-Light-Emitting Supramolecular Polymer Gel Based on *β*-CD and NDI Host-Guest Inclusion Complex

Srayoshi Roy Chowdhury, Sujay Kumar Nandi, Sahabaj Mondal, Santosh Kumar and Debasish Haldar *

Department of Chemical Sciences and Centre for Advance Functional Materials, Indian Institute of Science Education and Research Kolkata

Mohanpur 741246, West Bengal, India

E-mail: deba_h76@iiserkol.ac.in, deba_h76@yahoo.com

Table of Contents

1. Crystal Structure of NDI **1**…………………………….................................S3

2. Crystal Structure of NDI **2**…………………………………………….............S3

3. Packing diagram of NDI **2** ……………………………………………………….S4

4. POM image of β-CD in DMF……………………………………………………S4

5. Self healing property of organogel……………………………………………….S5

6. Synthesis of compounds **1-2**……………………………………………………...S6

7. Characterization of compounds **1-2**……………………………………….....S6

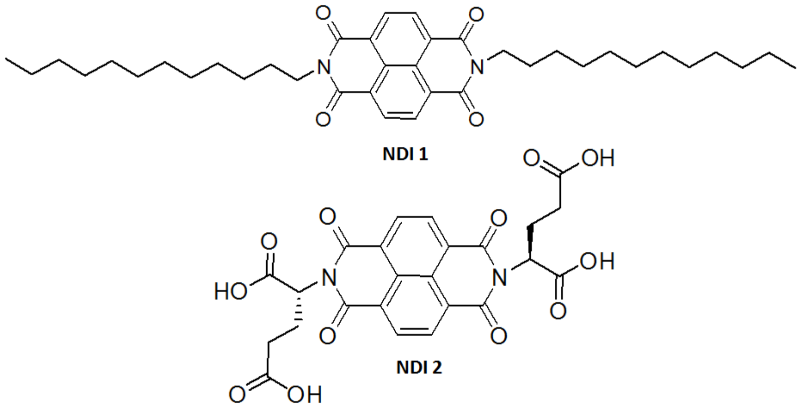

Scheme S1: Schematic presentation of NDI **1** and **2**.

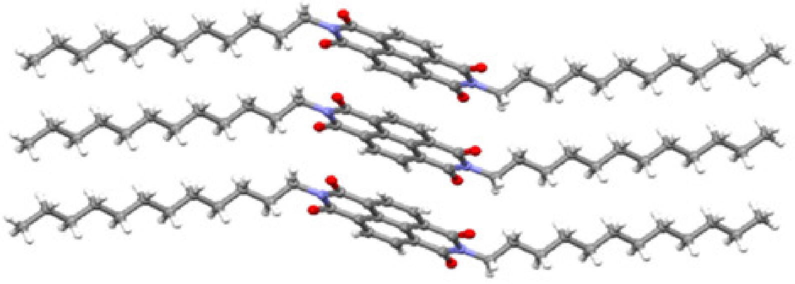

Figure S1: Higher order crystal structure of NDI **1** showing face-to-face π–π interactions between the central naphthalene diimide moieties.

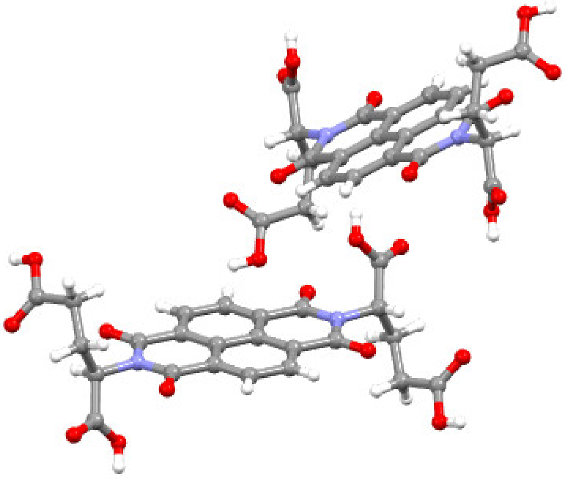

Figure S2: Crystal structure of NDI **2** showing absence of π-π stacking interaction.

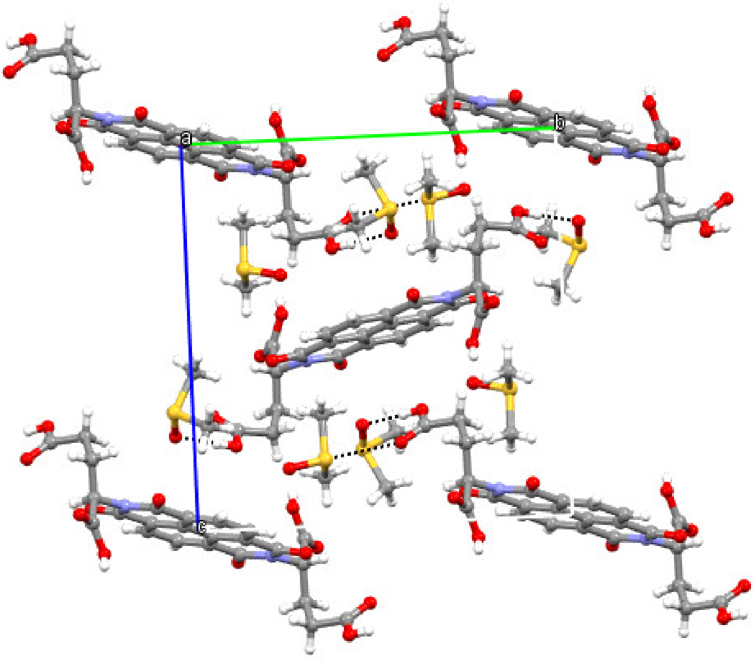

Figure S3: Packing diagram of NDI **2** along crystallographic *b* and *c* direction.

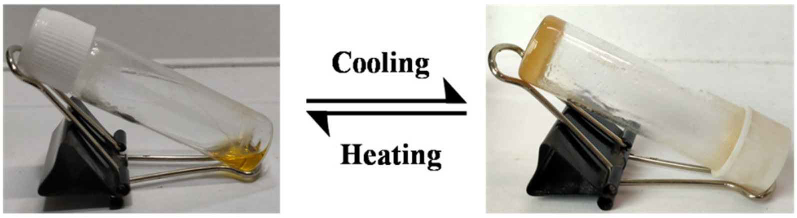

Figure S4: The supramolecular gel in DMF exhibit reversible sol to gel transitions induced by temperature at 66 °C.

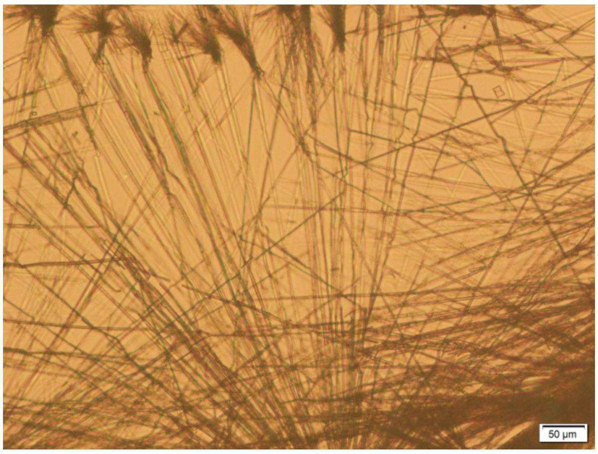

Figure S5: POM image of β-CD in DMF.

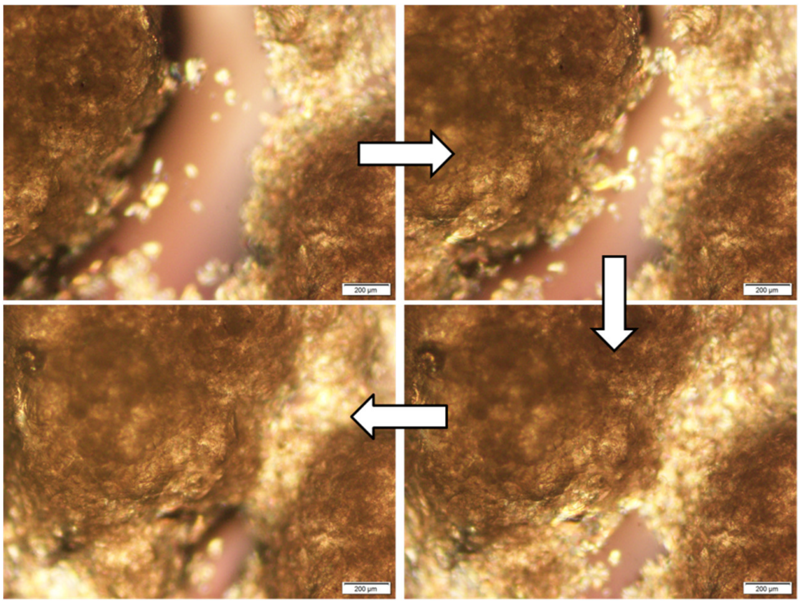

Figure S6: Self healing property of NDI **1** and β-CD in DMF gel by POM images.

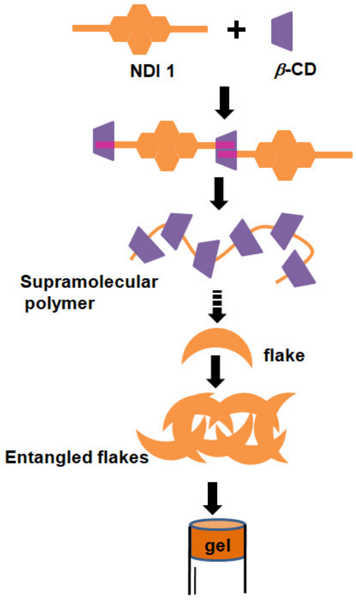

Figure S7: The schematic representation of the supramolecular polymer and gel formation.

Experimental:

Synthesis of compounds 1-2:

The NDI **1** and **2** were synthesized in a single step from commercially available naphthalenetetracarboxylicdianhydride, dodecyl amine and glutamic acid. The NDI **1** was synthesized by dissolving naphthalenetetracarboxylicdianhydride and dodecylamine in dry DMF and refluxed for 6 h with around 50% yield (as reported by Kozycz, L. M.; Guo, C.; Manion, J. G.; Tilley, A. J.; Lough, A. J.; Li, Y.; Seferos, D. S. Enhanced electron mobility in crystalline thionated naphthalene diimides. *J. Mater. Chem. C* **2015**, *3*, 11505–11515). The NDI **2** was synthesized by dissolving naphthalenetetracarboxylicdianhydride (5 mmol) and glutamic acid in glacial acetic acid and reflux for 6 h in ca. 30% yields. The mostly transparent solution was concentrated

under reduced pressure; 50 mL of DCM was added. For every case the organic layer was washed with 2 M HCl (3 x 50 mL), brine (2 x 50 mL) and dried over anhydrous Na_2SO_4 and evaporated under vacuum to obtain the corresponding NDI derivative. The crude product was purified by silica gel using hexane: ethyl acetate (1:2) as an eluent.

Characterization of NDI 1

Yield: 1.54g, (2.56 mmol, 51.2%). Off white colour solid, M.P. = 130–131°C.

^1H NMR (400 MHz, $CDCl_3$, δ in ppm, 298K): 8.98 (s, 4H, NDI aromatic protons), 4.20–4.16 (m, 4H, C^{α} H dodecyl), 1.76–1.69 (m, 4H, C^{β} H dodecyl), 1.41–1.27 (b, 36H, CH_2 dodecyl), 0.89–0.86 (m, 6H, CH_3 dodecyl); ^{13}C NMR (100 MHz, $CDCl_3$, δ in ppm, 298K): 160.89, 139.20, 128.67, 127.85, 125.47, 124.21, 41.77, 31.92, 29.39, 29.33, 28.04, 27.23, 22.77, 14.24. ESI-MS (MeOH): m/z (Calc): $C_{38}H_{54}N_2O_4Na$ $[M+Na]^+$ 625.1281; found: 625.1231.

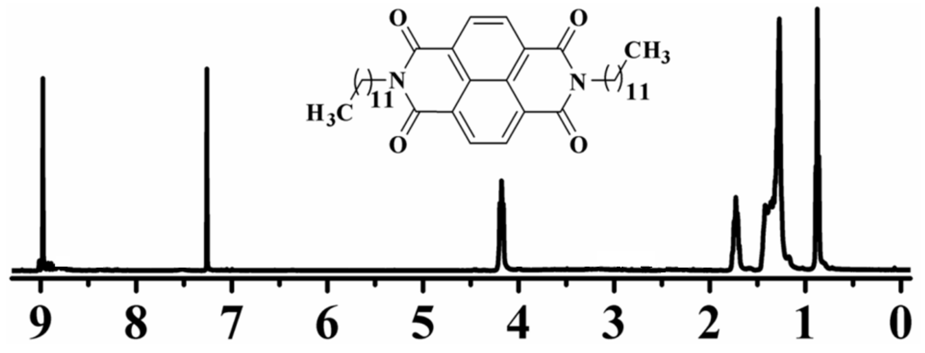

Figure S8: ^1H NMR (400 MHz, $CDCl_3$, δ in ppm, 298K) spectrum of NDI **1**.

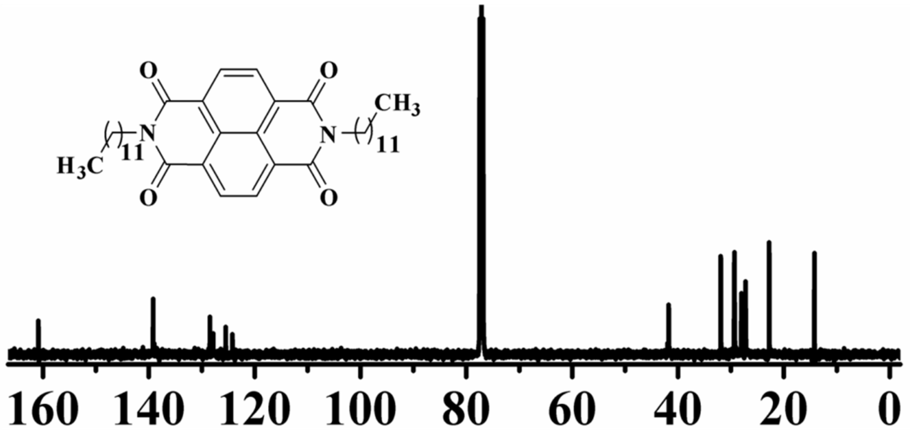

Figure S9: ^{13}C NMR (100 MHz, $CDCl_3$, δ in ppm, 298K) spectrum of NDI **1**.

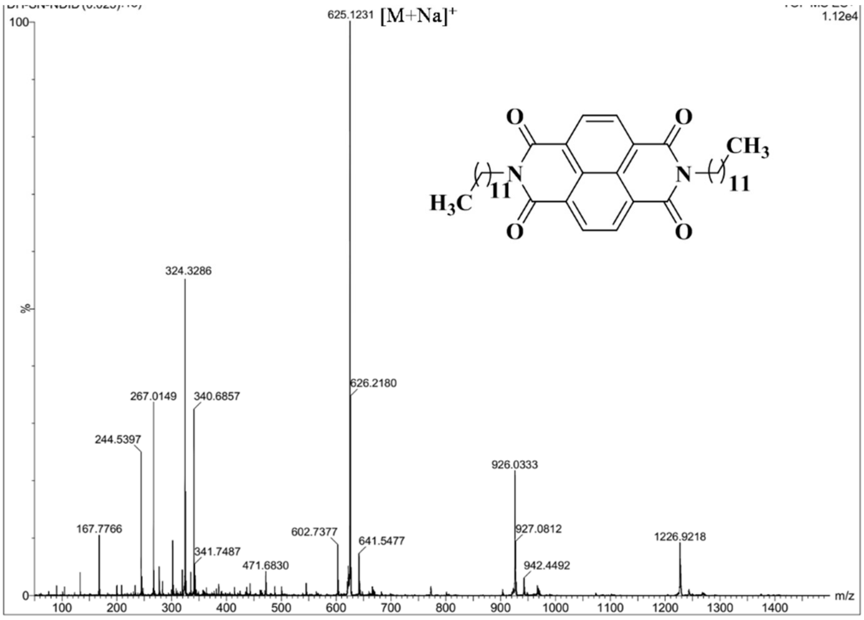

Figure S10: Mass spectrum of NDI **1**.

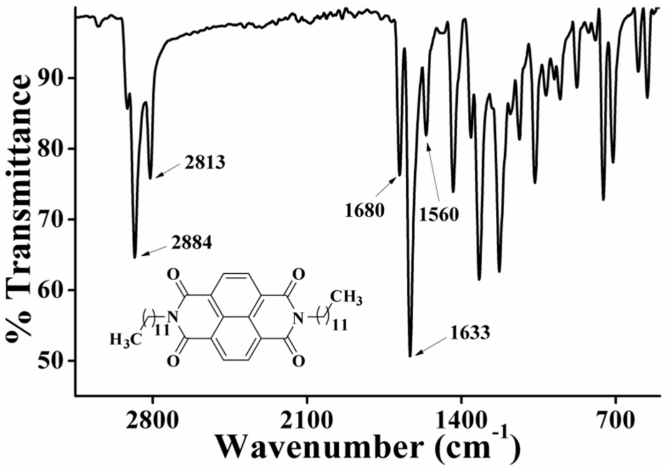

Figure S11: FT-IR spectrum of NDI **1**.

Characterization of NDI 2

Yield: 0.8g, (1.5 mmol, 30.5%). Brownish-yellow colour solid.

^{1}H NMR (500 MHz, DMSO-d$_6$, δ in ppm, 298K): 12.54 (b, 4H, Glu acid protons), 8.73 (s, 4H, NDI aromatic protons), 5.61–5.58 (m, 2H, C$^\alpha$ H Glu), 2.48–2.45 (m, 2H, C$^\gamma$ H Glu), 2.37–2.27 (m, 6H, , C$^\gamma$ H and C$^\beta$H Glu); ^{13}C NMR (125 MHz, DMSO-d$_6$, δ in ppm, 298K): 173.95, 170.41, 162.52, 131.09, 126.4, 126.12, 52.97, 30.53, 23.55. ESI-MS (MeOH): m/z (Calc): $C_{24}H_{17}N_2O_{12}NaK$ [M-H+Na+K]$^+$ 587.03; found: 587.0927.

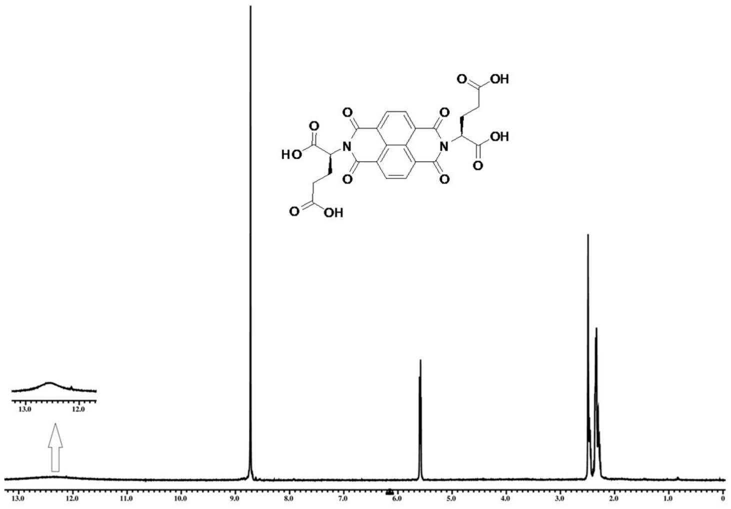

Figure S12: ^{1}H NMR (500 MHz, DMSO-*d*$_6$, δ in ppm, 298K) spectrum of NDI **2**.

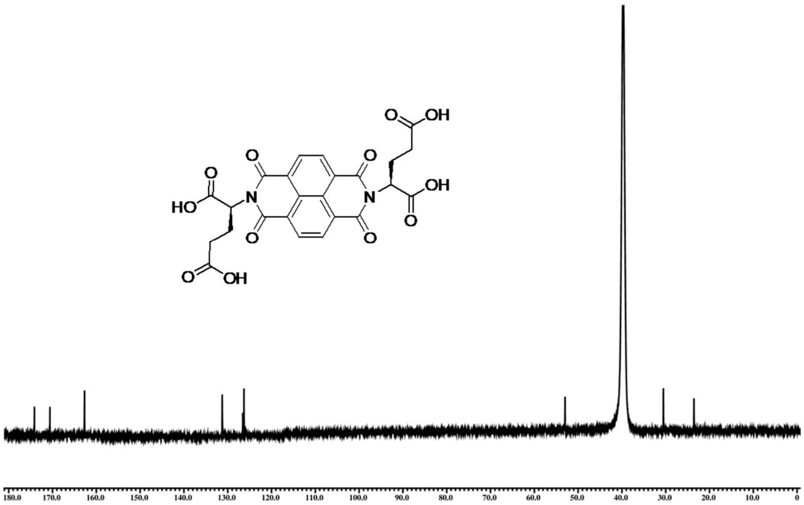

Figure S13: ^{13}C NMR (125 MHz, DMSO-*d*$_6$, δ in ppm, 298K) spectrum of NDI **2**.

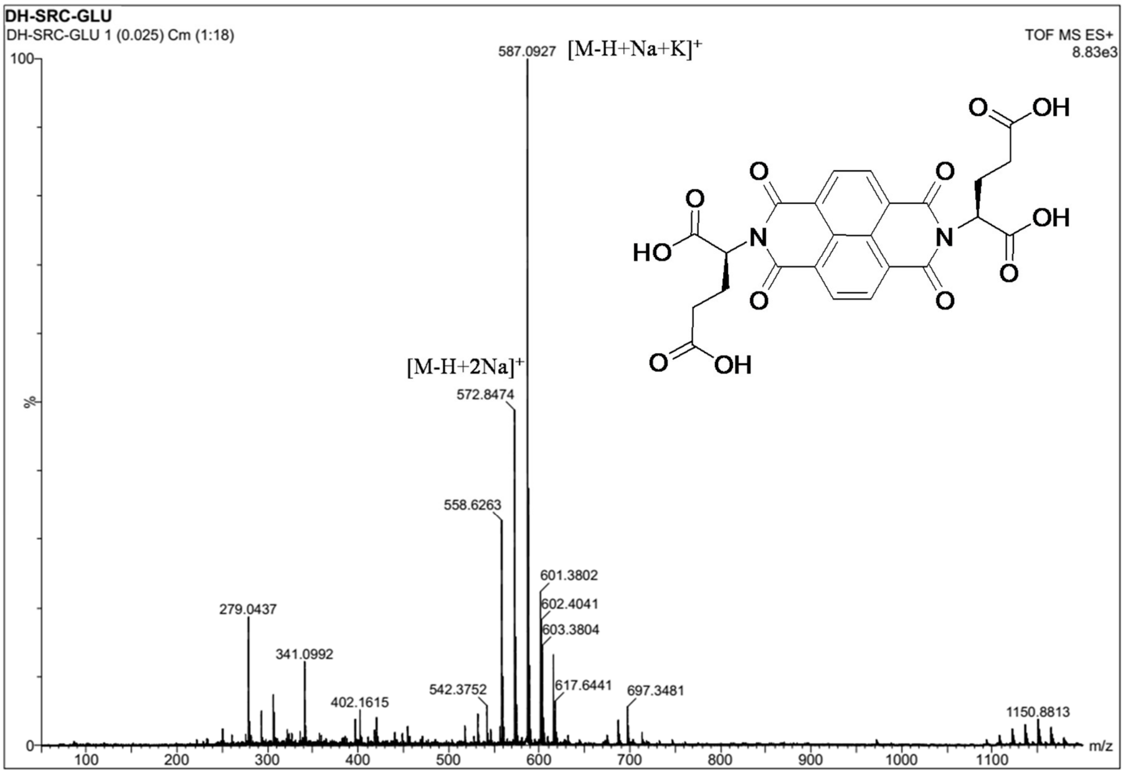

Figure S14: Mass spectrum of NDI **2**.

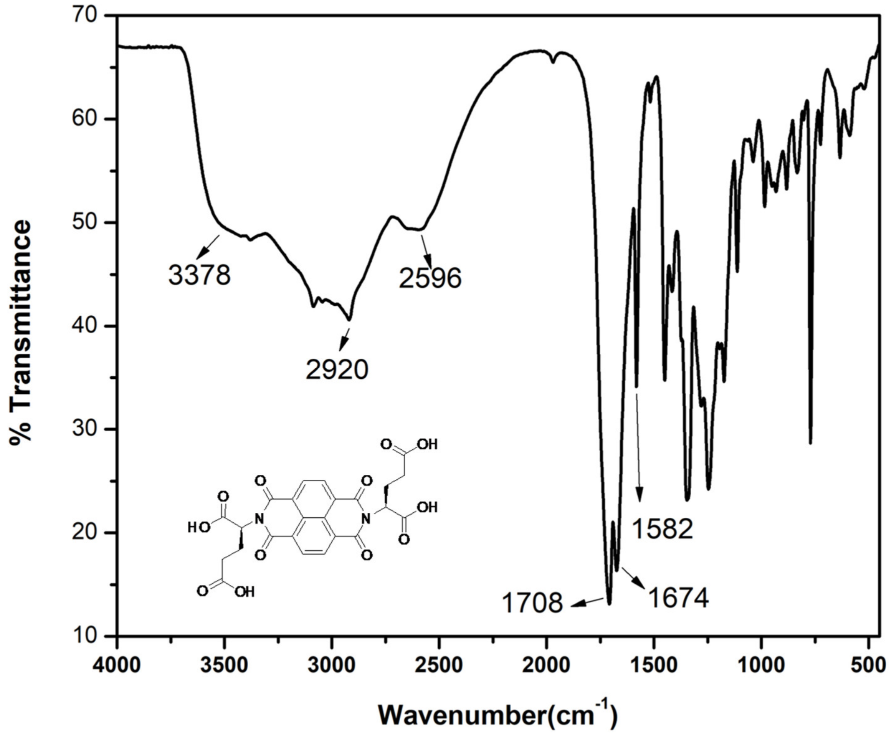

Figure S15: FT-IR spectrum of NDI **2**.
